# Synthesis of a Novel Hyperbranched Polyimide for Reinforcing Toughness and Insulating Properties of Bismaleimide Resin

**DOI:** 10.3390/polym14194234

**Published:** 2022-10-09

**Authors:** Lida Yu, Yang Yu, Jiahao Shi, Xiaorui Zhang, Feng Gao, Chenhao Li, Zhou Yang, Jingui Zhao

**Affiliations:** 1 Harbin University of Science and Technology, Harbin 150080, China; 2 Harbin Institute of Large Electrical Machinery, Harbin 150040, China; 3 State Key Laboratory of Hydropower Equipment, Harbin 150040, China; 4 Harbin Electric Machinery Company Limited, Harbin 150040, China

**Keywords:** hyperbranched polyimide, mechanical strength, impact strength, bending modulus, dielectric properties, glass transition temperature

## Abstract

Bismaleimide (BMI) resin has great potential in aerospace, electronic, and machinery fields due to its extraordinary thermal stability. Owing to BMI’s lower impact strength, various modified BMI resins have been prepared using CTBN, PEEK, fillers, and hyperbranched polymer to achieve higher impact strength. However, enhancement of toughness causes deterioration of other performance, such as Tg, thermal stability, and brittleness. In this work, BMI resin modified by hyperbranched polyimide (HBPI) was obtained. HBPI designed with flexible segments, unsaturated bonds, and a low degree of branching was synthesized. FT-IR and ^13^C-NMR were applied to confirm the successful fabrication of HBPI. The mechanical strength and dielectric properties of cured BMI resin containing various levels of HBPI were analyzed systematically. The impact and bending strength were improved significantly with increased HBPI content. When the content of HBPI is 40 wt.%, the impact strength and bending strength reach the maximum value of 32 kJ/mm and 88 MPa. In addition, the BMI cured with HBPI exhibits enhanced bending modulus to the value of 5.9 GPa. Furthermore, the dielectric strength of cured resin was improved to 28.3 kV/mm. The improved mechanical strength and enhanced dielectric properties are attributed to the increasing free volume induced by HBPI. These results indicate the promise of BMI resin modified by HBPI applied in insulating coatings and low dielectric laminates used in high frequency.

## 1. Introduction

Bismaleimide (BMI) resin is a kind of popular thermosetting resin, which is widely utilized in many advanced technical fields, such as aerospace, electronics, and machinery areas [1,2,3,4,5,6]. Although the thermal stability of BMI is excellent, lower impact strength caused by high crosslinking density and rigid network structure limits its further application.

Many studies have been carried out to improve the toughness of BMI resins, including modification by elastomeric polymer [7], thermoplastic polymer [8,9], hyperbranched polymer (HBP) [10], and filling nano-powders [11]. Modifying BMI resin with elastomeric polymer is an effective route to improve the impact strength; However, elastomeric polymer including rubber [12] and CTBN [13] cause a decrease in bending modulus and glass transition temperature. Thermoplastic polymers can both enhance BMI toughness and maintain modulus and thermal properties, making it the most widely used method for reducing the brittleness of BMI resin [14,15]. Some successful thermoplastic polymers with great thermal stability such as polyetherimides [16], poly(ether ketone)s [17] and poly(ether sulfone)s [18] have been introduced to BMI resin. However, increasing viscosity induced by blending with long-chain thermoplastic reduces the processing characteristics of BMI resin. Moreover, BMI resin reinforced by organic or inorganic filler also exhibits enhancing modulus and impact strength. Nano inorganic fillers, such as SiO_2_ [19] and TiO_2_ [20], and thermoplastic particles [21] have been tested in BMI resin, but the viscosity increased significantly with the addition of these fillers.

Hyperbranched polymers (HBP) employed to improve the mechanical strength of thermosetting polymers due to the unique spherical structure and multiple active end groups have been favored in recent years. The spherical structure of HBP can reduce the entanglement of molecules to retain processing characteristics [22,23] and provide free volume that can increase the movement space of molecular segments [24,25,26]. In addition, multiple active end groups of HBP improve the reactivity; they can react with matrix resin directly or be end-capped by functional molecules [27,28]. Therefore, BMI resin modified by HBP can achieve improved toughness [29,30]. Niu et al. [31] modified BMI resin using silicon-containing hyperbranched epoxy (SHBEp), which reacted with the BDM/DBA system through ring-opening reactions. The modified BMI resin system exhibited enhanced impact and bending strength when the content of SHBEp reached 8 wt.%. Wang et al. [32] used synthesized amino-terminated hyperbranched polysiloxane (HSiSn) as the organotin initiator to form Poly(butylene terephthalate) (PBT). The impact strength of modified PBT successfully increased with only 5 wt.% content of HSiSn/CBT. However, the bending modulus inevitably dropped due to the effect of the flexible segments. Since modulus and breakdown strength are influenced by the content of cluster free volume, enhanced modulus and dielectric properties could be obtained with a certain content of free volume. Shi et al. [33] used a newly synthesized hyperbranched unsaturated polyester molecule with flexible chains to improve the level of BMI properties. The results showed that the impact strength and flexural strength was increased by 211.1% and 209.3%, respectively, with an HBP mass of 20 wt.%.; the electronic properties were also enhanced significantly.

**Figure 1:**
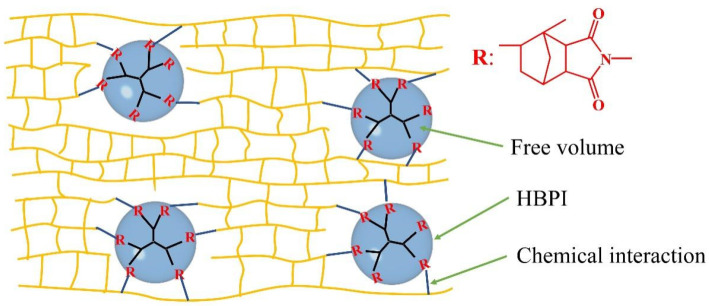
Cured network structure of HBPI modified BMI.

Although the enhanced toughness of BMI resin could be obtained, the modulus and dielectric properties of toughened BMI resin do not catch enough attention. In this paper, a novel hyperbranched polyimide (HBPI) with a low branching degree was designed and synthesized, and enhanced toughness of BMI modified with HBPI was achieved. The cured network of BMI modified with HBPI is shown in Figure 1. The structure of HBPI and cured BMI were analyzed systemically, and the mechanical and dielectric properties were investigated. The results present in this work suggest that HBPI with low branching degree modified BMI is an effective way to improve the toughness and breakdown strength of BMI resin while maintaining the mechanical modulus, which significantly explores the application of BMI in insulating coatings and low dielectric laminates used in the high-frequency area.

## 2. Experimental

### 2.1. Material

Hexaene Diisocyanate trimer (HDI trimer), Bisphenol A diallyl ether (BBE), and 2,2′-Diallyl bisphenol A (BBA) were purchased from Wanhua Chemical Group Co., Ltd., Yantai, China. All the compounds were used without further treatment. Methyl nadic anhydride (MNA), hydroquinone butyl acetate, and tert-butyl perbenzoate were obtained from Shanghai Macklin Biochemical Co., Ltd., Shanghai, China. Bismaleimide (BMI) was bought from Shanghai Aladdin Bio-Chem Technology Co., Ltd., Shanghai, China. The purity of all was analytical reagent (AR).

### 2.2. Preparation of Hyperbranched Polyimide and Cured Material

**Figure 2:**
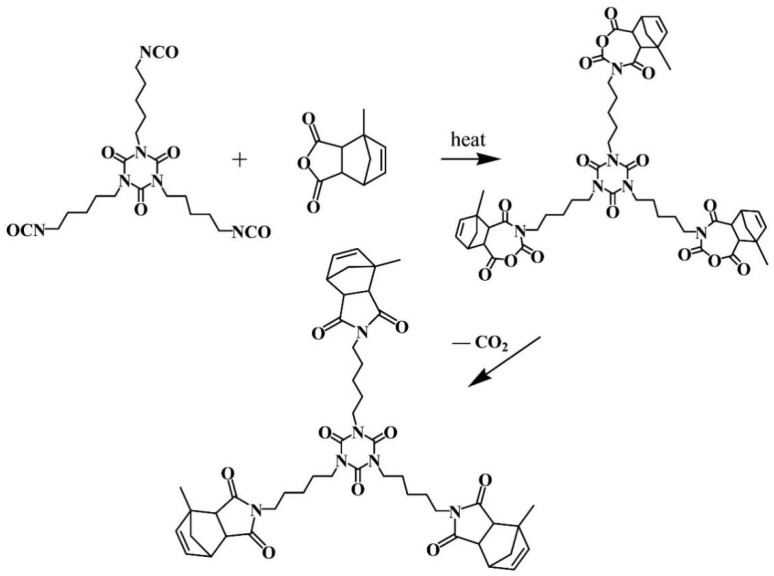
Synthesis reaction of HBPI.

The synthesized HBPI was derived from HDI trimer and Methyl nadic anhydride (MNA), which were used as core and blocking agents, respectively. The reaction is shown in Figure 2. The preparation is presented briefly below. HDI trimer, MNA, and hydro quinine were dissolved in butyl acetate in a three-necked flask, from which the molar ratio of HDI trimer to MNA was kept at 1:3, and the hydroquinine was 0.1 wt.% load of the system. Subsequently, the mixture was heated to 100 °C with stirring for 5 h under N_2_ atmosphere. After distilling butyl acetate with the suction filter, pure HBPI was obtained.

Modified BMI with various contents of HBPI were synthesized (contents of HBPI are 0 wt.%, 10 wt.%, 20 wt.%, 30 wt.%, 40 wt.%, 50 wt.%) with the same process, respectively. For example, the procedure of BMI modified with 40 wt.% HBPI is described as follows. BBA, BBE, and HBPI were mixed at 100 °C to result in a homogenous mixture. Then, BMI was added to the flask at 140 °C. The mixture was stirred until the mixture became transparent. The mole ratio of BBA, BBE, and BMI was 1:2:3. The weight amount of HBPI was 0.4 times that of the BBA/BBE/BMI system. After the degassing and cooling process, the mixture was poured into preheated teflon molds with the addition of 0.1 mol tert-butyl perbenzoate (TBPB). The curing cycle was 160 °C/1 h, 180 °C/1 h, 200 °C/1 h, 220 °C/1 h. The cured structure of the modified material is presented in Figure 2.

### 2.3. Instrumentation and Methods

^13^C-NMR characterization was performed by utilizing a 200 M superconducting ^13^C-NMR spectrometer produced by Varian (Palo Alto, CA, USA). The FT-IR spectra were recorded by EQUINOX55 (Brook, Germany) with wavenumber ranging from 500 cm^−1^ to 3500 cm^−1^. The number of FT-IR is 32. The FT-IR samples were made into liquid films that were sandwiched between KBr discs. The bending strength was conducted by using Instron universal testing machine (three-point bending test machine) at a moving rate of 1 mm/min with a 100 mm × 10 mm × 5 mm sample size. The specimen was placed on two supporting points at a certain distance, and a downward load was applied to the specimen at the midpoint of the two supporting points. The impact strength of samples (100 mm × 10 mm × 5 mm) were measured by XJJ-5 Impact Testing Machine which was manufactured by Chengde Testing Machine Co., Ltd. (Chengde, China). The dynamic mechanical analyses (DMA) results were obtained via TA Q800 dynamic mechanical analyzer (TA Instrument Company, New Castle, DE, USA) with a bending mode at a heating rate of 4 °C/min from 50 °C to 300 °C; all samples were tested with a vibration frequency of 1 Hz. The scanning electron microscope (SEM) images were conducted by JSM-7500F (Hitachi, Japan) with an accelerating voltage of 20 kV and all the samples received surface roughing treatment. The dielectric constant and dielectric loss were conducted by Alpha-A (German) with frequency ranging from 10 to 10^7^ Hz. The breakdown strength was tested using HT-100 (Guilin, China) in an oil environment with a spherical electrode type. Ten samples of each HBPI content with a size of 100 mm × 100 mm × 1 mm were tested with a voltage rate of 1 kV/s. Volume resistivity was 80, evaluated by EST121 (Digital Technology Co., Ltd. Beijing Hengrui Xinda, Beijing, China) with a measuring voltage of 1000 V. Thermogravimetric analysis (TGA) was conducted by utilizing Pyris 1 TGA (PerkinElmer, Waltham, MA, USA) at a heating rate of 5 °C/min from 50 °C to 600 °C, and with an N_2_ flow rate of 20 mL/min.

## 3. Results and Discussion

### 3.1. Characterization of HBPI

**Figure 3:**
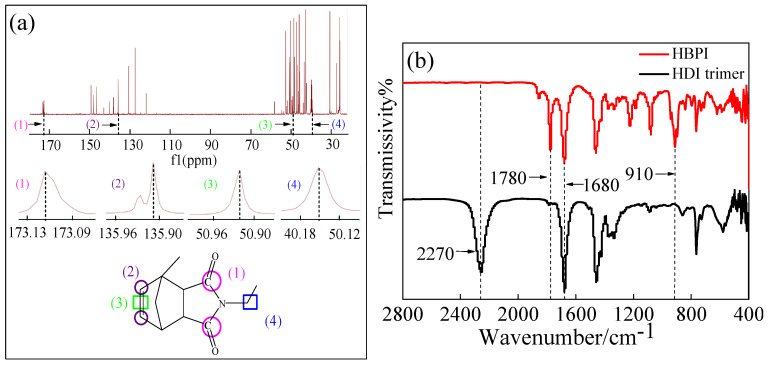
(**a**) ^13^C-NMR characterization of HBPI, (**b**) FT-IR characterization of HBPI and HDI trimer.

Figure 3a presents the ^13^C-NRM spectra of HBPI. The signal at the high field of 173.09~173.13 ppm can be attributed to the double bond with O, which can be assigned to carbon atoms marked as (1). In addition, the signals that appear in 135.9~135.96 ppm and 50.96~50.96 ppm can be assigned to carbon atoms marked as (2) and double bond moieties marked as (3). The signal of carbon atoms marked as (4) that connect N atom and carbon flexible chain appears in 40.18~50.12 ppm [34]. The results of ^13^C-NRM spectra indicate that HBPI with expected structure was synthesized successfully. The FT-IR spectra are shown in Figure 3b. For HBPI, the peak at 2270 cm^−1^ which was attributed to -N=C=O of HDI disappeared after the HBPI was synthesized, which indicated the successful reaction between isocyanic and anhydride. The characteristic stretching peak of imide -C=O at 1780 cm^−1^ appears in the upper band indicating the presence of imide rings that derive from MNA. Furthermore, the peak of HBPI at 910 cm^−1^ proves the existence of double bonds, while there is no peak appearing at 1780 cm^−1^ and 910 cm^−1^ in the HDI trimer band [34,35]. The results show ^13^C-NRM spectra and FT-IR spectra demonstrating that HDI trimers have reacted with NMA, and the resultant of reaction is HBPI.

### 3.2. Mechanical Property of Cured Materials

**Figure 4:**
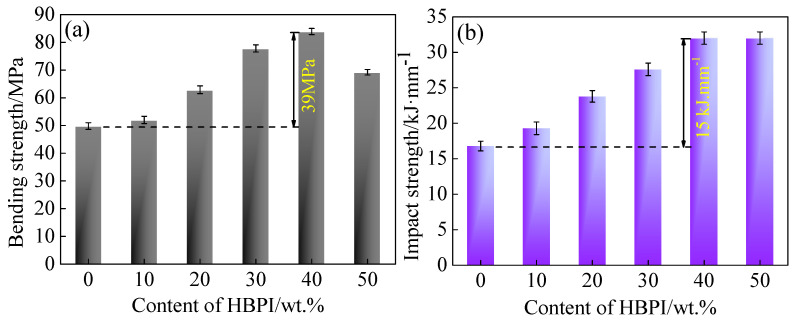
Mechanical properties of HBPI modified BMI resin with various content of HBPI. (**a**) bending strength, (**b**) impact strength.

The bending strength of modified BMI with various levels of HBPI content are presented in Figure 4a. The bending strength increases with HBPI content, and reaches the maximum value of 89 MPa, which is 79.6% higher than unmodified BMI, when the content of HBPI is 40 wt.%, then decreases with increasing HBPI content. Since the residual internal stress and crosslinking density of cured BMI could be reduced by free volume and flexible segments, which are induced by HBPI, the bending strength can be improved with increased HBPI content. When the content of HBPI is higher than 40 wt.%, bending strength decreases due to the excess free volume, which reduces the rigidity of the BMI.

The impact strength of all samples was investigated and the results are presented in Figure 4b. The impact strength significantly increases with increasing content of HBPI. The maximum value of 32 kJ/mm is obtained in cured BMI with 40 wt.% HBPI, which is 88.2% larger than unmodified BMI. Moreover, the impact strength decreases slightly with HBPI content further improving. A possible mechanism is proposed here as follows. On the one hand, free volume and flexible segments induced by HBPI could absorb the impacting energy. On the other hand, free volume with certain added content may decrease the crosslinking density, resulting in the enhancement of impact strength. As a result, the impact strength is improved with increased HBPI content. However, excessive free volume would severely reduce the crosslinking density of cured BMI, causing the degradation of toughness [36]. Therefore, the enhanced impact strength is obtained in modified BMI with certain levels of HBPI.

In addition, it is worth noting that degradation of impact strength from 40 wt.% to 50 wt.% of HBPI was much smaller than that of bending strength. The probable reason is as follows. The impact strength (toughness) of BMI depends on the stress and strain that a material experiences when it breaks. With the further increasing of HBPI, the bending strength was decreased because of the degradation of rigidity, causing a negative impact of toughness. However, the excessive flexible segments induced by HBPI may have increased the deformation at break, offsetting the negative effect of bending strength.

**Figure 5:**
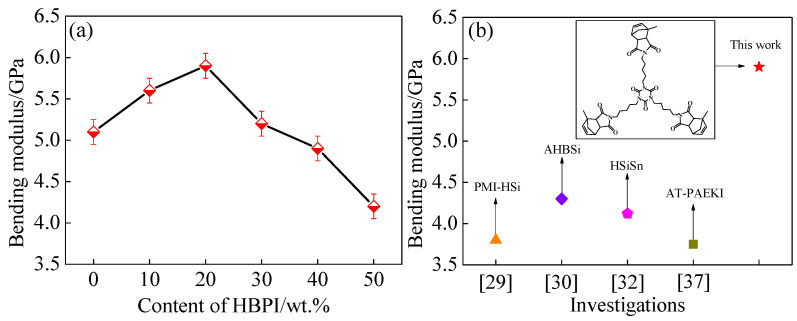
(**a**) Bending modulus of HBPI modified BMI resin with different content of HBPI, (**b**) Comparison of modified BMI with 20 wt.% of HBPI and previous investigations.

The bending modulus testing results of modified BMI with various levels of HBPI content are shown in Figure 5a. The bending modulus was slightly improved when the content of HBPI was lower than 20 wt.%. The maximum value of 5.9 GPa was obtained in BMI with 20 wt.% HBPI, which was 15.7% higher than unmodified BMI. The bending modulus decreased significantly with increased HBPI content. Although increasing content of the flexible segments reduced the bending modulus, the imide ring induced by HBPI and chemical interaction between HBPI and BMI enhances the bending modulus [37]. Moreover, the addition of HBPI leads to the dispersion of small fractured free volumes and offsets the negative effect of free volume on the modulus, due to the low branching degree of HBPI. As a result, the bending modulus of modified BMI is improved with certain levels of HBPI.

Maintaining the modulus while improving the toughness of BMI is always the key problem of BMI modification. Figure 5b compares the bending moduli from previous methods that used different hyperbranched polymers with high degrees of branching to modify BMI. Hyperbranched polysiloxane is a kind of common HBP to toughen BMI [29,30,32]. The moduli of these hyperbranched polysiloxane modified BMI material were 97~102% of neat BMI, indicating a slight change. Utilizing hyperbranched polyimide [37] (AT-PAEKI) significantly enhanced the modulus with relatively low content, due to the imide rings and chemical interaction between AT-PAEKI and BMI. However, as the content of AT-PAEKI increased, clustered free volume decreased the stiffness. Correspondingly, in this work, the changing trend of modulus is similar to AT-PAEKI modified BMI material. Nevertheless, the modulus was also kept well with even a large amount of HBPI. The explanation is that HBPI with a low degree of branching enabled small free volumes to uniformly scatter in the BMI resin, leading a stiffer performance of HBPI modified materials.

### 3.3. Dynamic Thermomechanical Analysis

**Figure 6:**
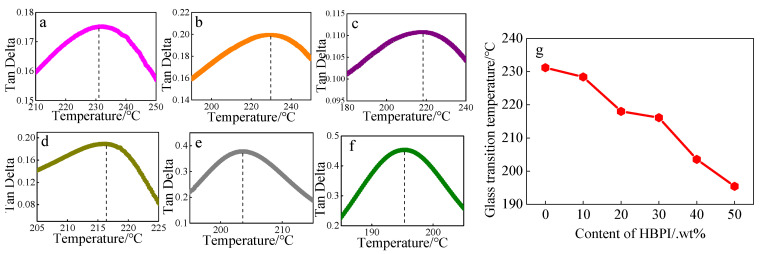
The DMA results of BMI and modified BMI with HBPI. (**a**–**f**) Neat BMI and modified BMI with 10%, 20%, 30%, 40%, 50% amount of HBPI, (**g**) Changing trend of Tg of modified BMI with different HBPI content.

The glass transition temperatures (T_g_) of BMI and modified BMI were measured using DMA and the results are shown in Figure 6a. The T_g_ of resins gradually decreases with HBPI content increasing. As increasing free volume induced by the spherical structure of HBPI provides the conditions for movement of chain segments, easier movement of chain segments leads the reducing of T_g_. Thus, the BMI with higher HBPI content exhibits lower T_g_. Although a higher content of HBPI has an adverse effect on the thermal property of modified BMI, modified BMI still performs well in thermal stability permitting a work temperature of 200 °C.

### 3.4. Microstructure of BMI Resin Modified with HBPI

**Figure 7:**
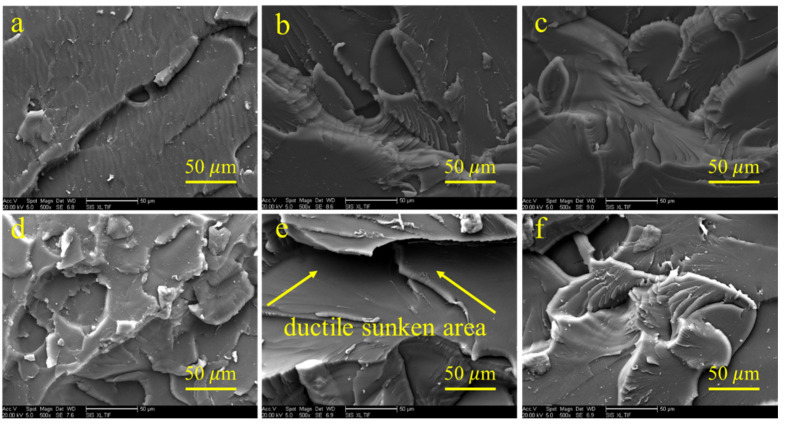
The morphology of the fractured surface of unmodified BMI and HBPI modified BMI after impact test. (**a**) pure BMI, (**b**–**f**) modified BMI with 10 wt.%, 20 wt.%, 30 wt.%, 40 wt.%, 50 wt.% HBPI.

The morphology of fractured surfaces was studied by SEM images, which are presented in Figure 7. The impact fracture surface of unmodified BMI shown in Figure 7a is relatively flat. And the cracks were unidirectional, which indicates that the process of cracks propagation was not affected by resistance force. The impact fracture surface of unmodified BMI exhibits typical features of brittle fracture. The impact fracture surface becomes rough with increased HBPI content, as shown in Figure 7b–f. The appearance of the ductile sunken area indicates that the free volume successfully absorbed the impact energy and diverted the cracks. Therefore, the impact fracture surface of modified BMI exhibits characteristics of tough fracture.

### 3.5. Insulating Performance of Cured Materials

**Figure 8:**
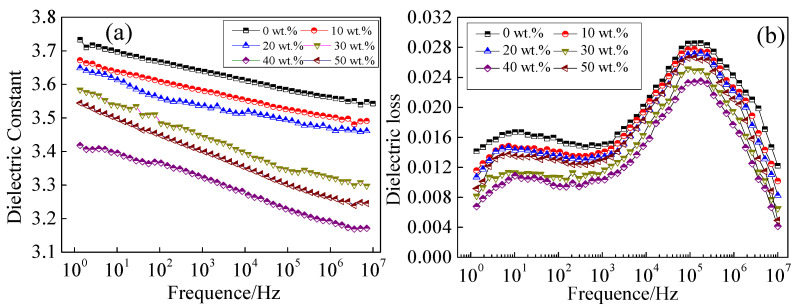
Dielectric property of modified BMI with different content of HBPI. (**a**) dielectric constant, (**b**) dielectric loss.

The dielectric properties of cured BMI modified by HBPI were investigated and the results are shown in Figure 8. The dielectric constants of the samples are presented in Figure 8a. The dielectric constant decreases with increasing content of HBPI, and reaches a minimum value of 3.2 at 10^7^ Hz when the content of HBPI is 40 wt.%. The dielectric constant is influenced by the density of polar groups; hence, the increased content of free volume induced by HBPI causes reduced density of polar groups. Moreover, HBPI exhibits a low dielectric constant due to the symmetrical molecular structure. Thus, the dielectric constant decreases with increased HBPI content. However, the dielectric constant of BMI increases again when the content of HBPI is larger than 40 wt.%. Because the higher content of free volume reduces the crosslinking density of curing BMI, the orientation polarization formed by the remaining small molecules is established more easily under an applied electric field. As a result, the dielectric constant increases with larger HBPI content.

Figure 8b shows the dielectric loss of cured BMI with various contents of HBPI. The dielectric loss decreases with increasing HBPI content, and reaches a minimum value at 40 wt.%. As crosslink sites provided by HBPI have the ability to promote the curing process of BMI, the content of defects induced by the curing process decreases with increased HBPI content, which causes the decrease of dielectric loss. Nevertheless, excessive HBPI obviously decreases the crosslinking density of cured BMI. Lower crosslinking density provides easier molecular vibration, which leads the increasing of dielectric loss of modified BMI with the higher levels of HBPI. Thus, the dielectric loss rises when HBPI content is larger than 40 wt.%.

**Figure 9:**
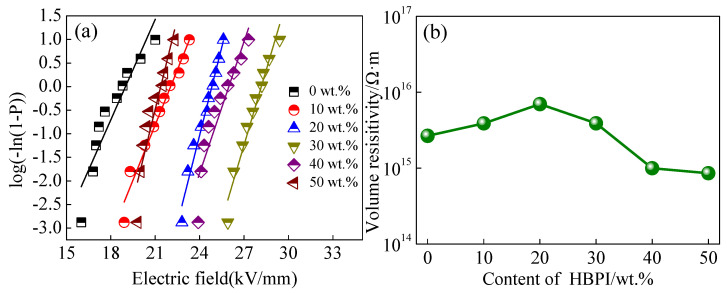
Insulation performance of modified BMI with various levels of HBPI. (**a**) breakdown strength, (**b**) volume resistivity.

The breakdown strength of cured BMI with various contents of HBPI were measured and the fitting results are shown in Figure 9a. The breakdown strength obviously increases with increased content of HBPI. The maximum value of 28.3 kV/mm is obtained when the content of HBPI is 30 wt.%. The reduction of concentration of defects in cured BMI resin due to the presence of HBPI leads to a decreased distance between BMI molecular chains, which hinders the formation of electron avalanches by movement of the initial electrons. Thus, the addition of HBPI improves the breakdown strength of modified BMI resin. When the content of HBPI is larger than 30 wt.%, the breakdown strength decreases with increased HBPI content. Since excess free volume induced by HBPI causes loosening of the crosslink network, free carriers can move easily under an applied electric field. As a result, the breakdown strength decreases as HBPI content increases.

Figure 9b presents the volume resistivity of all samples. The volume resistivity of BMI increases slightly with increased content of HBPI, and the maximum value is obtained in BMI with 20 wt.% HBPI. Because the content of defects in the basic resin can be reduced by HBPI, the molecular chains of the modified BMI resin become closer. Decreasing distance between molecular chains limits the movement of free carriers, which results in the enhancement of volume resistivity. When the content of HBPI is higher than 40 wt.%, the volume resistivity significantly decreases. The excessive free volume induced by the spherical structure of HBPI lessens the network of molecular chains of cured resin, and the weakened network intensifies the movement of free carriers. Therefore, the volume resistivity decreases with higher HBPI content.

### 3.6. Thermal Property of Modified BMI Resin

**Figure 10:**
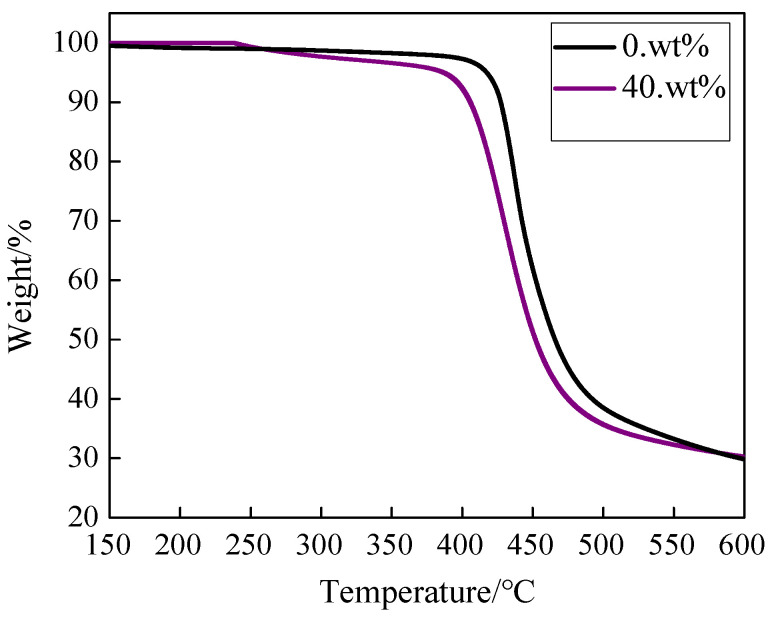
Thermogravimetric curves of modified BMI with different content of HBPI.

Thermogravimetric analysis curves of BMI and modified BMI are presented in Figure 10. The decomposition temperature of modified BMI resin is 400 °C, which is lower (25 °C) than that of unmodified BMI. However, the thermal stability of modified BMI can still meet the needs of a high temperature environment that relate to insulating coatings and low dielectric laminates used in the high-frequency area. The modified BMI has a lower decomposition temperature due to the presence of HBPI. On the one hand, the soft segments in HBPI can be decomposed at lower temperature. Moreover, the residuals remaining in the HBPI system during the preparation process, such as methyl nadic anhydride and butyl acetate, cause the modified BMI resin to lose weight at lower temperatures. On the other hand, a higher content of HBPI reduces the crosslinking density of modified BMI, which also decreases the decomposition temperature. Although the decomposition temperature of BMI modified with HBPI is lower, the modified BMI resin exhibits excellent insulating properties, mechanical strength, and thermal stability simultaneously.

## 4. Practical Implemention

The HBPI was added mainly to improve the performance of neat BMI. The excellent thermal stability and insulating properties could enable BMI to be qualified for insulating coating or low dielectric laminates used in the high frequency area. However, it is very common for materials in these areas to experience shock stress. Obviously, the brittle neat BMI can not satisfy this condition. In the present investigation, HBPI is shown to significantly improve the toughness and mechanical strength of BMI. In addition, the special characteristics of HBPI offset the negative effect of free volume and maintain the mechanical modulus, satisfying the requirement of laminates in terms of modulus.

## 5. Conclusions

In the study, a novel HBPI with a low degree of branching and imide rings was successfully synthesized, and was employed to enhance the mechanical strength of BMI resin.

1. The structure of HBPI was successfully confirmed by FT-IR and NMR.
2. The mechanical properties of the modified BMI were significantly improved. The impact strength reached 32 kJ/mm. The bending strength increased up to 88 MPa.
3. The modulus was retained while the toughness and mechanical strength were improved.
4. The enhanced insulating properties of modified BMI resin were obtained with a certain level of HBPI. The breakdown strength reaches 30 kV/mm when the content of HBPI is 30 wt.%.
5. The heat resistance of modified BMI was still satisfactory and meet sthe need of insulating coatings and low dielectric laminates used in the high-frequency area.

The improved performance of modified BMI is due to the designed structure of HBPI. The imide ring of HBPI contributed to enhancing the modulus of modified BMI, and the low branching degree of HBPI was responsible for the improvement of the breakdown strength by dispersing free volume more efficiently.

Above all, BMI materials with different contents of HBPI have different performance characteristics. The content of HBPI can be regulated to adapt to specific situations or demands. These results indicate that modified BMI with HBPI is an effective route to broadening the application of BMI resin, such as for insulating coatings and low dielectric laminates used in the high frequency area. The idea of synthesizing HBP with low degrees of branching may provide direction and guidance for maintaining the modulus of BMI while improving the toughness.
